# Zero-Field NMR of Urea: Spin-Topology Engineering by Chemical Exchange

**DOI:** 10.1021/acs.jpclett.1c02768

**Published:** 2021-10-27

**Authors:** Seyma Alcicek, Piotr Put, Danila Barskiy, Vladimir Kontul, Szymon Pustelny

**Affiliations:** † Institute of Physics, Faculty of Physics, Astronomy and Applied Computer Science, Jagiellonian University in Kraków, 30-348 Kraków, Poland; ‡ Helmholtz Institute Mainz, GSI Helmholtz Center for Heavy Ion Research GmbH, 55128 Mainz, Germany; ¶ Institute of Physics, Johannes Gutenberg-Universität, 55128 Mainz, Germany

## Abstract

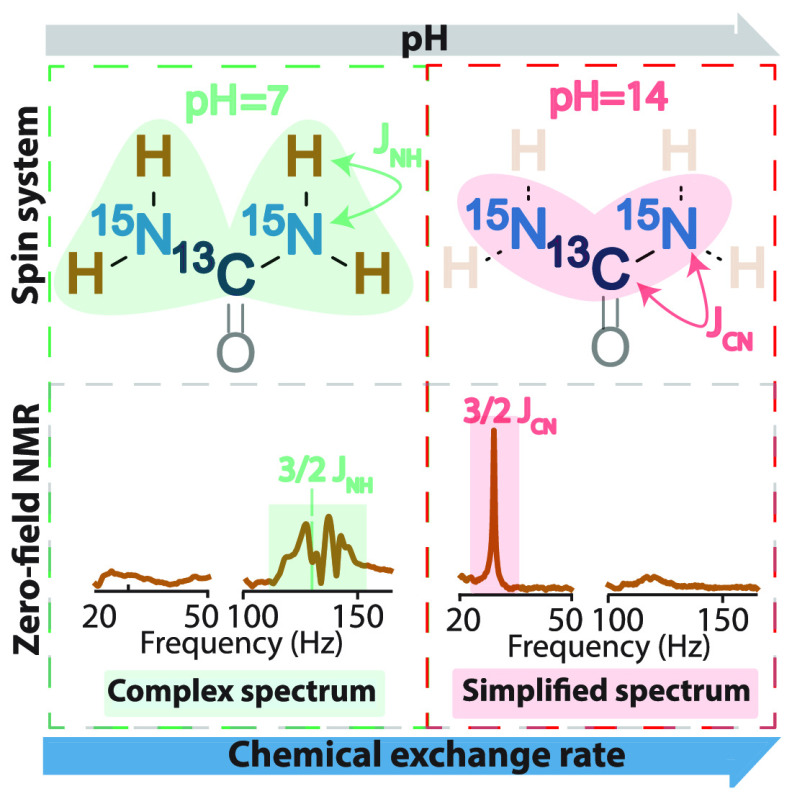

Well-resolved and information-rich *J*-spectra are the foundation for chemical detection in zero-field NMR. However, even for relatively small molecules, spectra exhibit complexity, hindering the analysis. To address this problem, we investigate an example biomolecule with a complex *J*-coupling network—urea, a key metabolite in protein catabolism—and demonstrate ways of simplifying its zero-field spectra by modifying spin topology. This goal is achieved by controlling pH-dependent chemical exchange rates of ^1^H nuclei and varying the composition of the D_2_O/H_2_O mixture used as a solvent. Specifically, we demonstrate that by increasing the proton exchange rate in the [^13^C,^15^N_2_]-urea solution, the spin system simplifies, manifesting through a single narrow spectral peak. Additionally, we show that the spectra of ^1^H/D isotopologues of [^15^N_2_]-urea can be understood easily by analyzing isolated spin subsystems. This study paves the way for zero-field NMR detection of complex biomolecules, particularly in biofluids with a high concentration of water.

Zero- and ultralow-field (ZULF) nuclear magnetic resonance (NMR) is a novel, portable, and cost-effective technique that enables high-precision chemical analysis through direct observation of intramolecular spin interactions at ultralow (typically <100 nT) external magnetic field.^1−6^ Because in isotropic liquids direct magnetic dipolar and quadrupolar interactions average out to zero, under the zero-field regime, an electron-mediated, indirect spin–spin coupling (also known as *J*-coupling) becomes the dominant interaction.^7^ This allows the use of zero-field NMR for the determination of a whole *J*-coupling network in the molecule and hence chemical fingerprinting.^8,9^ Because chemical exchange alters spin–spin couplings and NMR relaxation rates, ZULF NMR is capable of monitoring this process, involving chemical reactions (bond-breaking and bond-making) or conformational modifications (bond rotation), as was shown in a recent study.^10^ The application of ZULF NMR was recently demonstrated in the context of biomolecules consisting of 2–5 coupled nuclear spins.^6,11^ However, for larger spin systems, ZULF NMR spectra become complicated because of the increased number of coupled nuclei, making the spectral analysis challenging. Here, we present various approaches for modifying and simplifying zero-field spectra of molecules containing a large number of spins, some of which undergo a chemical exchange. For this purpose, we use ZULF NMR *J*-spectroscopy to investigate solutions of urea, a molecule with a large coupling network and exchangeable protons.

Urea is an important biomolecule, which plays a vital role in amino acid and protein metabolism, enabling 80–90% of nitrogen excretion from the human body. It is produced in the liver through the urea cycle, transported via the bloodstream, and excreted into urine by the kidneys.^12^ Therefore, measuring the urea level in urine and blood is a routinely used medical diagnostic technique to evaluate liver and kidney function.^13−15^ Moreover, [^13^C]-urea and [^13^C,^15^N_2_]-urea have recently become attractive contrast agents for hyperpolarized magnetic resonance imaging studies, as urea is a highly biocompatible and valuable marker for the evaluation of myocardial perfusion and renal physiology.^16−18^ Finally, the interest in urea is also stimulated by a growing demand for robust and reliable compound detection in fields such as environmental monitoring,agricultural and food chemistry.^19^

In our work, we investigate [^15^N_2_]-urea and [^13^C,^15^N_2_]-urea in various solution environments by observing changes in the zero-field NMR *J*-spectra. First, we demonstrate the influence of the proton exchange process on spectra by measuring [^15^N_2_]-urea and [^13^C,^15^N_2_]-urea in an aprotic solvent, dimethyl sulfoxide (DMSO), and a protic solvent, water (H_2_O). Because the proton exchange rate in urea is both acid- and base-catalyzed, we then investigate aqueous solutions of urea at various pH levels. The results are explained by zero-field NMR simulations, considering the combined effect of chemical exchange and nuclear spin dynamics using a simple theoretical model. [^15^N_2_]-urea was also measured in the mixtures of H_2_O and D_2_O to study the effect of deuterium nuclei on the zero-field *J*-spectra. The experimental results are supported by simulations taking into account the proportion of ^1^H/D isotopologues of urea in solution. All spectral peaks, arising from *J*-coupling interactions (^15^N–^1^H, ^15^N–D, and ^1^H–D) in spin subsystems, are identified by analyzing the energy-level structures of isotopologues using perturbation theory. On the basis of the presented results, we show straightforward ways to study complex biomolecules with ZULF NMR by taking advantage of the chemical exchange process.

The urea molecule contains two −NH_2_ groups joined by a carbonyl (C=O) functional group. To analyze the general structure of *J*-spectra of [^15^N]-urea and [^13^C,^15^N_2_]-urea, first, numerical simulations are performed using *J*-coupling constants shown in Figure 1.^20,21^ Because one-bond ^1^H–^15^N coupling in the −NH_2_ group is the strongest interaction in this system, the main features in the *J*-spectra of both forms of urea are centered around (3/2)|^1^*J*_NH_| ≈ 133.65 Hz (marked by a dashed line in Figure 1), as expected for an XA_2_ nuclear spin system corresponding to the transitions in the manifold with the total proton spin 1 (see, for example, refs (8) and (22)). Hereafter, we refer to this group of peaks as high-frequency peaks. Other (weaker) homonuclear and heteronuclear interactions result in the appearance of low-frequency peaks (<10 Hz) as well as further modifications (splitting and shifting) of the high-frequency peaks. Specifically, the presence of an additional, relatively strong ^13^C–^15^N interaction in [^13^C,^15^N_2_]-urea increases the shifts in corresponding energy manifolds, giving rise to a wider span of low- and high-frequency peaks compared to the spectrum of [^15^N_2_]-urea.

**Figure 1:**
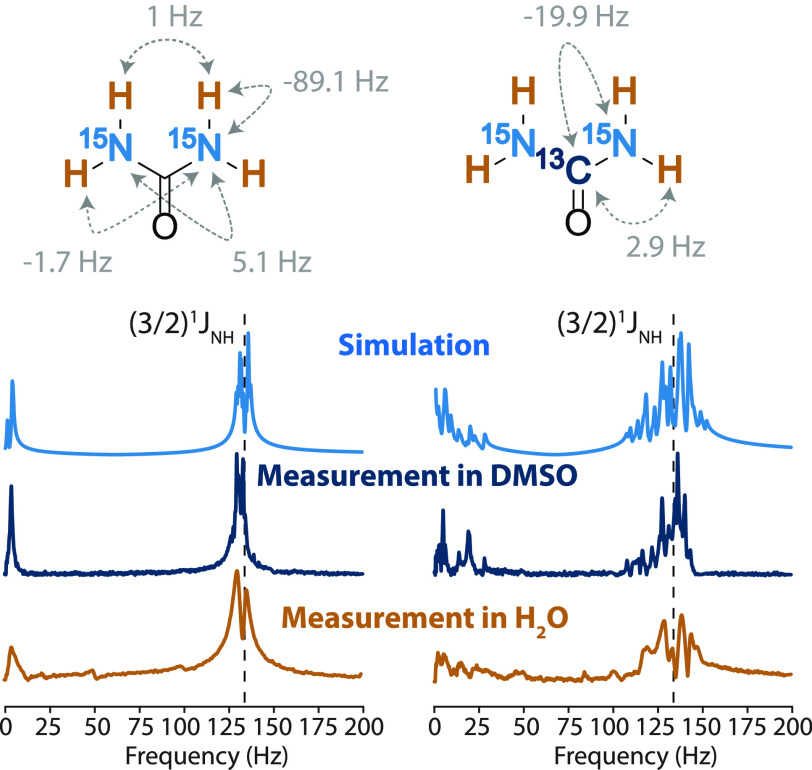
Simulated and experimental ZULF NMR spectra of [^15^N_2_]-urea and [^13^C,^15^N_2_]-urea in aprotic (DMSO) and protic (H_2_O) solvents. The structural formulas are shown with *J*-coupling values used in the simulation.

In Figure 1, *J*-spectra of [^15^N]-urea and [^13^C,^15^N_2_]-urea in dimethyl sulfoxide and water are compared. The experimental spectra of urea in DMSO agree well with the simulation, especially in terms of peak positions. The lines become substantially broader (approximately 3 times) when water is used as a solvent (Figure 1). This effect is expected because amide protons are known to undergo chemical exchange with water protons, and this process contributes to the nuclear-spin relaxation rate.^23^ However, the mere fact of being able to observe these multiplets in *J*-spectra indicates that the proton exchange rate in urea at a neutral pH level is slow enough for ZULF NMR measurements, compared to the spin evolution originating from *J*-couplings. This is also confirmed by examining the chemical exchange rate for urea in neutral pH, being equal to approximately 1.9 s^–1^, which is significantly slower than the dominant interaction in the system (^1^*J*_NH_).^23^ Furthermore, because of the absence of heteronuclear spin–spin coupling, the water signal contributes only to a peak at 0 Hz at truly zero magnetic field, which does not overlap with *J*-spectra of target molecules. This feature makes zero-field NMR a promising modality for the analysis of biological samples with a high concentration of water (e.g., blood, urine, or cell cultures) because there is no need for solvent suppression.^24^

The proton exchange process in aqueous solutions of urea is pH-dependent and both acid- and base-catalyzed. Here, we distinguish two exchange processes:^25−28^
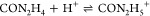
1
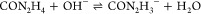
2In this part of the work, the effect of the proton exchange rate on *J*-spectra is studied by varying pH of the solution while maintaining the same concentration of urea (8 M). As shown in Figure 2, because of the increased proton exchange rate in urea solutions, the amplitudes of high-frequency peaks (120–150 Hz) gradually decrease without considerable line broadening at both low and high pH values. It is clear that, when the proton exchange rate is much higher than the *J*-coupling (*k*_ex_ ≫ *J*_NH_), ^1^H nuclei are effectively decoupled from the rest of the spin system and the *J*_NH_-coupling does not contribute to the observed zero-field spectra. Therefore, high-frequency peaks vanish in the spectra of highly acidic (pH 1.4) and highly basic (pH 14) solutions, which is also supported by simulations of the urea spin system (Figure 2; see also Methods). The disappearance of the high-frequency peaks under highly acidic/basic conditions also bears a resemblance to the results shown in a recent study on zero-field NMR of ammonium in highly acidic conditions.^10^ The authors of ref (10) reported that an increase in the proton exchange rate causes the zero-field NMR signals of ammonium to vanish. This is explained by the nature of the experiment: after prepolarization in a strong field, a sample spends a significant amount of time (1 s) in a low-field region (tens of μT) before being detected in zero field. In our experiment, we are limited to a shuttling time (time between prepolarization and signal acquisition) of 1 s, because for the shorter transfer times, vibration noise, stemming from a NMR-tube transport, disrupts the structure of the spectra. For such a delay, water protons depolarize despite a guiding field of 10 μT. In the case of a faster proton exchange, unpolarized protons are more often involved in the exchange process. This affects the “memory” of nuclear spin orders, resulting in the reduction of amplitudes of peaks. To verify the influence of the guiding field strength on the peaks’ amplitudes, the field was increased by an order of magnitude, which, because of the increased proton relaxation time *T*_1_, resulted in an up to 25% signal enhancement (Figure S1).^29^ The increase of the signal amplitude in the stronger guiding field is predicted to be universal for molecules under the rapid chemical exchange and can be exploited in measuring zero-field spectra of such molecules. The boost in the signal, stemming from stronger transfer field, may not only be beneficial for remote prepolarization experiments but also find use in ZULF hyperpolarization techniques, which rely on the chemical exchange;^30,31^ stronger magnetic field slows down relaxation of protons in solution, which yields higher signal amplitudes of zero-field NMR signals.

**Figure 2:**
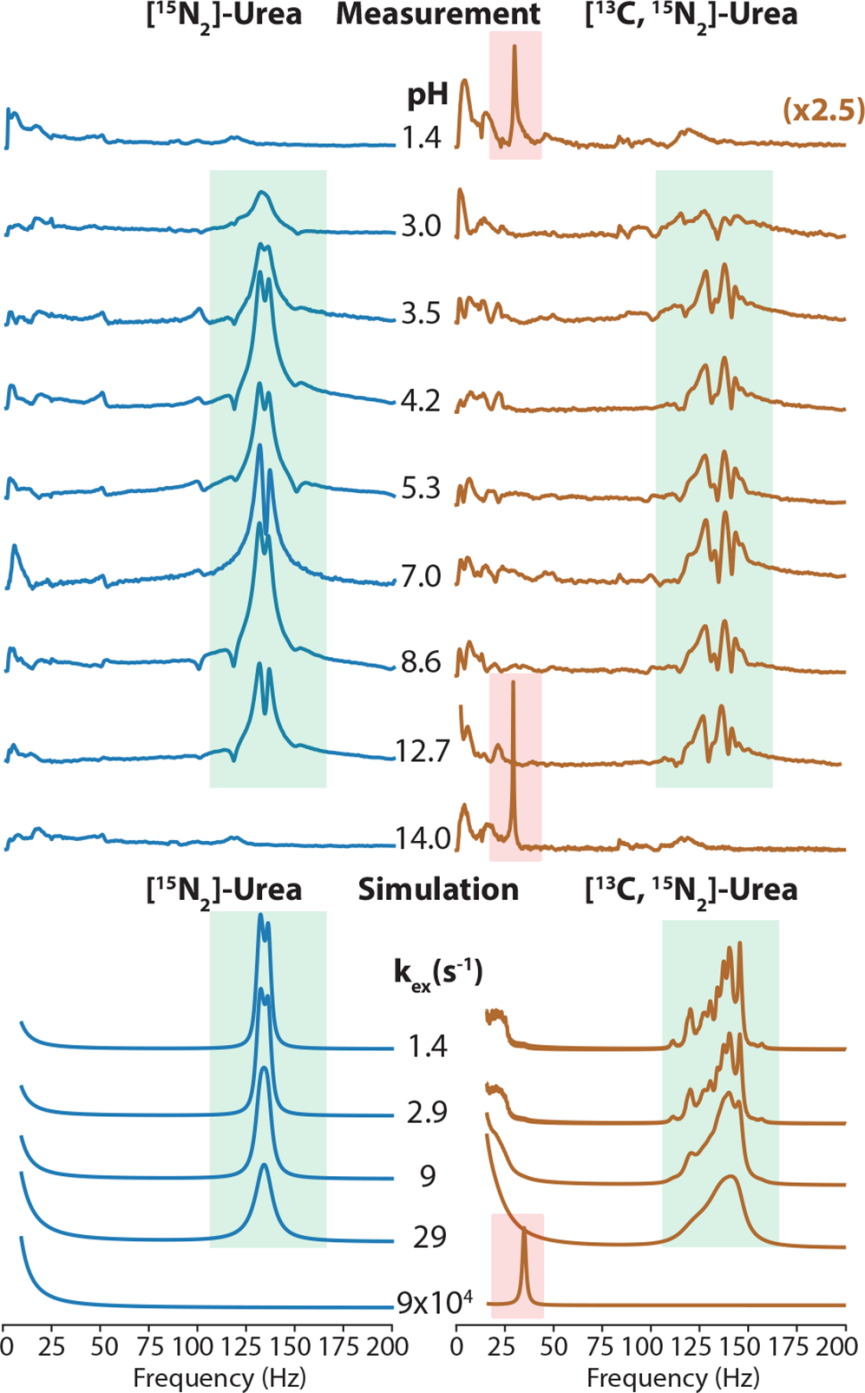
Experimental (top) and simulated (bottom) zero-field *J*-spectra of [^15^N_2_]-urea and [^13^C,^15^N_2_]-urea in aqueous solutions at various pH values. The peaks arising from one-bond, strong *J*-coupling interaction between ^15^N and ^1^H are green shaded (120–150 Hz), while the narrow peaks (around 30 Hz), originating from one-bond *J*-coupling between ^13^C and ^15^N, are highlighted in red.

On the other hand, a rapid proton exchange leads to the modification of the effective spin system, which can greatly simplify the observed spectra. This is demonstrated in the spectra of [^13^C,^15^N_2_]-urea in highly acidic and basic solutions (red boxes in Figure 2), where a narrow peak appears close to 30 Hz. This signal arises at (3/2)*J*_CN_ and originates from the *J*-coupling between ^13^C and ^15^N nuclei in the CN_2_ spin system, where, because of the rapid exchange, the protons are effectively decoupled from the rest of the nuclei. The emergence of the low-frequency peak is also supported by the numerical simulations for the spin system under rapid chemical exchange (shown in the bottom of Figure 2). It should be stressed that, by taking advantage of the accelerated chemical exchange, a narrower single peak (1 Hz width) with higher amplitude arises in the zero-field spectrum because of a modification of the spin topology from the complex XAB_2_A′B′_2_ system to the simple XA_2_ system. This simplified spectrum, however, still depends on the molecule-specific combination of *J*-coupling strength and coupling pattern, enabling the chemical fingerprinting.

Next, we investigate ^1^H/D isotopologues of urea. For this study, we modify the spin coupling network, replacing ^1^H (spin-1/2) with deuterium (spin-1), by dissolving urea in D_2_O/H_2_O mixture. To simulate *J*-spectra of urea solutions with various D_2_O/H_2_O ratios, the proportion of each isotopologue in solution is calculated using a binomial distribution. Because a probability of an amide-proton site being occupied by deuterium depends on the fraction *p* of deuterium in the solution, the molar fraction *x* of each isotopologue is given by:
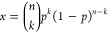
3where *n* is the number of possible sites where deuterium nuclei can reside and *k* is the number of deuterium nuclei that each isotopologue contains. Simulated spectra of all isotopologues are next summed after weighing each spectrum with an appropriate binomial coefficient. The D–^15^N coupling constants are estimated using the appropriate *J*_NH_ constants and the gyromagnetic ratios of deuterium and proton, where the *J*_ND_ coupling constant is equal to *J*_ND_ ≈ (γ_D_/γ_H_)*J*_NH_. This approach neglects secondary isotope effects.^32^ As a result, we obtain a good agreement between the experimental spectra of urea solutions with the various ratios of D_2_O/H_2_O and their simulated counterparts (Figure 3).

**Figure 3:**
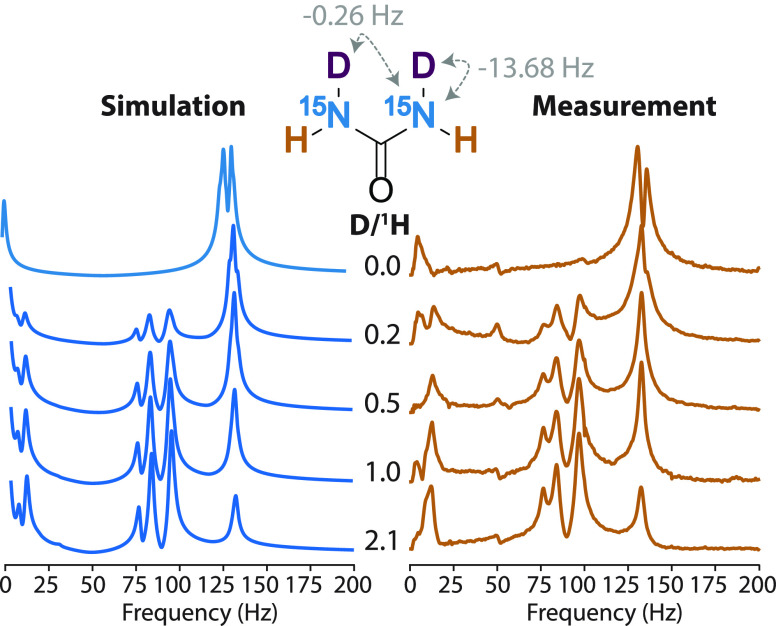
Experimental and simulated zero-field *J*-spectra of [^15^N_2_]-urea in aqueous solutions with various ^1^H/D ratios. *J*-coupling values used in simulations are shown with chemical structures of an example ^1^H/D isotopologue of urea. All isotopologues’ structures and corresponding simulated zero-field *J*-spectra are shown in the Supporting Information.

In the analysis of *J*-spectra of urea ^1^H/D isotopologues, two nitrogen atoms are treated as equivalent. Hence, the isotopologues consist of three different spin subsystems: −NH_2_, an XA_2_ spin system; −NHD, an (XA)B spin system; and −ND_2_, an XB_2_ spin system. As shown in Figure 4, the peaks arising from −NH_2_ and −NHD groups are predicted using the first-order perturbation theory. It should be also noted that signals from the −ND_2_ group are not observed in the spectra. This results from the fact that the relative amplitude of the ZULF NMR signal is proportional to the square of the difference between gyromagnetic ratios of *J*-coupled nuclei,^7^ which equals  ≈ 0.0025 (see the Supporting Information for the detailed energy-level analysis).

**Figure 4:**
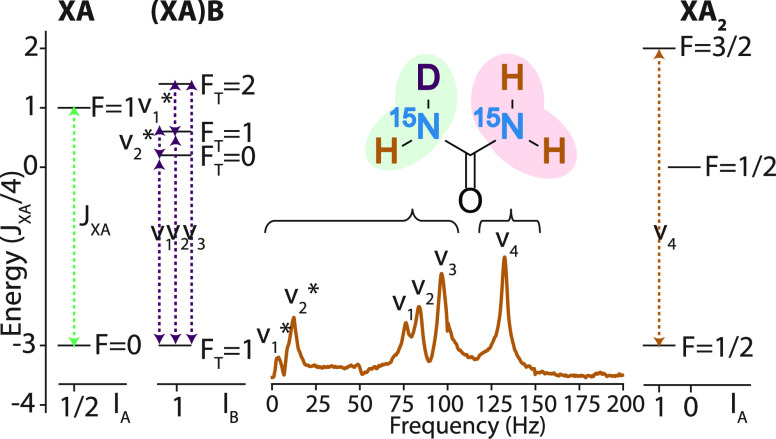
Left and right: Energy-level structures for XA, (XA)B, and XA_2_ spin subsystems. High and low-frequency transitions in (XA)B spin system are denoted by v_1–3_ and , respectively. The transition in XA_2_ spin system is represented as v_4_ which corresponds to 3/2*J*_XA_. The manifolds are grouped by the quantum numbers *I*_A_ and *I*_B_ that denote the spin number of A nuclei and B nuclei, respectively. Each manifold is labeled by its total spin quantum number *F* or *F*_T_ (see Supporting Information for detailed energy level analysis).^5,9^ Only a single sublevel in each manifold and a single transition at each frequency are shown for clarity. Middle: Experimental spectra of [^15^N_2_]-urea in the mixture of D_2_O/H_2_O (1:1). For all peaks in the spectrum, the corresponding transitions (v_1–4_, ) are determined by the first-order perturbation theory.

The observation of quadrupolar nuclei (spin > 1/2) in zero-field NMR is challenging because of their additional electric influence on reorientation of nuclei that can cause fast relaxation.^29^ Previous studies show that even though the *J*-couplings to deuterium, ^14^N, and ^35/37^Cl nuclei may not be directly visible as peaks in zero-field spectra, they may cause additional line-broadening.^5,33^ Conversely, in the study on zero-field NMR of quadrupolar nuclei, peaks originating from *J*-coupling interactions of ^1^H–D and ^1^H–^14^N are shown in *J*-spectra of ammonium isotopologues.^34^ Because of a relatively small electric moment of deuterium and a high local symmetry of ^14^N-ammonium resulting in small nuclear quadrupolar interactions, the detection of *J*-coupling interactions of such nuclei in zero-field NMR is feasible.^29^

Our results demonstrate that zero-field NMR is able to detect ^1^H/D isotopologues of urea molecules as well as provide information on ^1^H/D ratio in solution through simulation of *J*-spectrum. We also show that *J*-spectra for the complex molecules with more than two heteronuclei can be interpreted clearly by analyzing the energy structure of each small spin subgroup separately.

To summarize, we investigated urea, one of the crucial biomolecules, under various solution conditions using ZULF NMR. We demonstrate that the compound can be readily detected in water by modifying spin topology under the chemical exchange process. Our results can be extrapolated to other biomolecules with similar structures (e.g., amino acids), facilitating various biochemical research. We also report that the *J*-spectra of complex molecules, such as urea isotopologues, can be clearly interpreted by identifying simple subgroups in the system and analyzing their energy structures independently. All the experimental results are congruent with simulations, confirming our theoretical interpretation. This work could enable future *in vivo*/*in vitro* investigations of complex biomolecules. Such studies might be possible in biofluids (e.g., blood, urine, *etc*.) with a high concentration of water. Specifically, one of the significant clinical analysis methods, the quantification of urea in urine and blood, will be a subject of our future research. However, in the presented study, we worked with highly concentrated (5–8 M), isotopically enriched urea solutions. Even with such high concentrations, low thermal prepolarization, provided by the 1.8 T magnet, results in weak ZULF NMR signals. To overcome this limitation, zero-field NMR can be combined with hyperpolarization methods such as parahydrogen-induced polarization (PHIP),^35,36^ signal amplification by reversible exchange (SABRE),^37^ dynamic nuclear polarization (DNP),^10^*etc*. However, because these methods are limited to just a selection of molecules, the more universal exchange-based polarization methods such as SABRE-RELAY and PHIP-X may be preferable for a diverse set of biomolecules.^30,31^

## Methods

All chemicals were purchased from Sigma-Aldrich and used without further purification. [^13^C,^15^N_2_]-urea (CAS# 58069-83-3) and [^15^N_2_]-urea (CAS# 2067-80-3) solutions at various pH values were prepared in an 8 M concentration by dissolving in sodium hydroxide (CAS# 1310-73-2) or hydrochloric acid (CAS# 7647-01-0). The pH of each sample was measured at room temperature using a portable pH meter (Mettler Toledo Seven2Go) with a micro electrode (Mettler-Toledo InLab Pro-ISM). For the study of the ^1^H–D exchange, 8 M [^15^N_2_]-urea solutions were prepared by dissolving urea in distilled water, D_2_O (CAS# 7789-20-0), and 25%, 50%, and 75% distilled water–D_2_O (CAS# 7789-20-0) mixtures. For preparation of urea solutions in an aprotic solvent, [^13^C,^15^N_2_]-urea (CAS# 58069-83-3) and [^15^N_2_]-urea were dissolved in DMSO (CAS# 67-68-5) with a final concentration of 5.4 M. Each sample (0.15 mL) was placed inside a standard 5 mm NMR tube and then flame-sealed under vacuum (<10^–4^ mbar) following degassing by several freeze–pump–thaw cycles.

The NMR samples are thermally polarized for 20 s using a 1.8 T magnet placed above the magnetic shield and mechanically shuttled into the zero-field detection region (inside a magnetic shield), where the magnetic field of the nuclear spins is measured using a home-built alkali-vapor atomic magnetometer. During the transfer, lasting roughly 300 ms (plus an additional 700 ms delay), a guiding field of 10 μT is applied by a solenoid wrapped along the whole length of the shuttling path. When the sample reaches the detection area, the guiding field is turned off suddenly to generate an oscillating NMR signal.^7^ Each zero-field NMR spectrum is the result of averaging 2048 transients. The entire data processing is performed using Python. A comprehensive description of the experimental setup and a detailed explanation of data processing can be found in ref (5).

A high-performance spin simulation library Spintrum is employed to simulate zero-field NMR spectra through numerical diagonalization of density matrices describing the spin systems.^3,38^ The *J*-coupling values in the simulations are taken directly from the literature or estimated using the gyromagnetic ratios of nuclei (see discussion above). Simulations of chemical exchange effects on the zero-field spectra of urea were obtained using an approach presented in ref (10). Details of the calculations as well as a discussion of possible shortcomings of the used exchange model are discussed in the Supporting Information.

## Supporting Information Available

Dependence of urea zero-field NMR signal amplitude on the guiding field strength; measured ZULF NMR spectra in deuterated urea; simulated spectra of deuterated urea isotopologues; and details of chemical exchange simulations in zero-field (PDF)

## Notes

The experimental data will be provided upon request.
