# Supplementary material for: Zero-Field NMR of Urea: Spin-Topology Engineering by Chemical Exchange
Source: J Phys Chem Lett. 2021 Oct 27;12(43):10671–6. doi: 10.1021/acs.jpclett.1c02768 (PMC8573776; doi:10.1021/acs.jpclett.1c02768)
Supplement: Supplementary file 1 — jz1c02768_si_001.pdf [file jz1c02768_si_001.pdf]

Supplementary Information: Zero-field NMR of Urea: Spin-Topology Engineering by Chemical Exchange

Seyma Alcicek,[1,*] Piotr Put,[1] Danila Barskiy,[2,3] Vladimir Kontul,[1] and Szymon Pustelny[1,†]

[1] *Institute of Physics, Faculty of Physics, Astronomy and Applied Computer Science, Jagiellonian University in Kraków, 30-348 Kraków, Poland*

[2] *Helmholtz Institute Mainz, GSI Helmholtz Center for Heavy Ion Research GmbH, 55128 Mainz, Germany*

[3] *Institute of Physics, Johannes Gutenberg-Universität, 55128 Mainz, Germany*

A. The amplitude of urea's zero-field NMR signal versus the strength of transfer field

Depolarization of chemically exchanging protons during shuttling from a prepolarizing magnet to a zero-field region leads to the decrease of the zero-field NMR signals. To investigate the process, we applied a magnetic field along the shuttling path and vary its value from 0.01 mT to 0.1 mT[1] (Fig. S1). As shown in Fig. S1, increasing the field by an order of magnitude results in a roughly 25% increase in the signal amplitude. In this way, we verified that guiding field strength has a significant influence on the amplitude of the zero-field NMR signal of molecules under the chemical exchange.

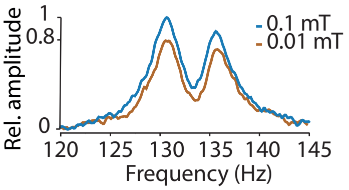

FIG. S1. Zero-field J-spectra of $[^{15}N_2]$-urea solutions measured for shuttling fields of 0.01 mT and 0.1 mT. The signals are acquired after the field is switched off.

* seyma.alcicek@uj.edu.pl

† szymon.pustelny@uj.edu.pl

B. Frequencies of resonance lines in deuterated urea J-spectra

To interpret zero-field spectra of molecules containing more than two equivalent nuclei, the perturbation theory was employed successfully in a few studies[2–5]. For a nuclear spin system with three heteronuclei (XA)B, the total nuclear spin Hamiltonian $\hat{\mathcal{H}}$ at zero field can be written as:

$$\hat{\mathcal{H}} = J_{\mathrm{XA}}\,\hat{\mathbf{S}}\cdot\hat{\mathbf{I}}_A + J_{\mathrm{XB}}\,\hat{\mathbf{S}}\cdot\hat{\mathbf{I}}_B + J_{\mathrm{AB}}\,\hat{\mathbf{I}}_A\cdot\hat{\mathbf{I}}_B, \tag{S1}$$

where $\hat{\mathbf{S}}$, $\hat{\mathbf{I}}_A$, $\hat{\mathbf{I}}_B$ are the spin vector operators corresponding to the spin X, A, and B, respectively. When J_{XA} coupling is a dominant interaction in the system, the unperturbed energies of the levels, defined by three quantum numbers S, I_A, F, are given by:

$$E = \frac{J_{\mathrm{XA}}}{2}\left[F(F+1) - I_A(I_A+1) - S(S+1)\right], \tag{S2}$$

where F ranges from $|I_A - S|$ to $I_A + S$ in steps of unity. The additional (weaker) interactions J_{XB} and J_{AB} cause the energy-level shifts, given by:

$$E^1 = \frac{J^1_{\mathrm{XB}} + J^1_{\mathrm{AB}}}{2}\left[F_T(F_T+1) - F(F+1) - I_B(I_B+1)\right], \tag{S3}$$

where $\hat{\mathbf{F}}_T$ is a total-spin operator ($\hat{\mathbf{F}}_T = \hat{\mathbf{I}}_B + \hat{\mathbf{F}}$) with F_T being a total-spin quantum number ranging from $|I_B - F|, |I_B - F| + 1, ..., I_B + F - 1, I_B + F$, and J^1_{XB}, J^1_{AB} represent scaled J-coupling constants:

$$\begin{aligned} J^1_{\mathrm{XB}} &= \frac{J_{\mathrm{XB}}}{2}\left[1 + \frac{S(S+1) - I_A(I_A+1)}{F(F+1)}\right], \\ J^1_{\mathrm{AB}} &= \frac{J_{\mathrm{AB}}}{2}\left[1 + \frac{I_A(I_A+1) - S(S+1)}{F(F+1)}\right]. \end{aligned} \tag{S4}$$

To find observable transitions between the shifted energy levels, we employ following selection rules: $\Delta I_A = \Delta I_B = 0, \Delta F_T = 0, \pm 1$,[3]. This procedure allows us to approximately determine the position of the split lines. In this work, we present detailed results of the first-order perturbative calculation for the (NH)D spin subsystem in partially deuterated urea. The values of all observable transitions are presented in Table S1.

C. Simulated spectra of deuterated urea isotopologues

We simulated the zero-field J-spectra of individual urea ^{1}H-D isotopologues (for chemical structures see insets in Fig. S2) by a numerical diagonalization of corresponding density matrices using the `Spintrum` package in Python[8]. As shown in Fig. S2, each of the isotopologues

TABLE S1. Allowed transition frequencies (ν) between levels characterized with the quantum numbers F and F_T, obtained using first-order perturbation theory, in the (XA)B spin subsystem. The last column gives the numerical values of the corresponding transitions in (NH)D, using J-coupling constants of J_{NH}= 89.1 Hz, J_{ND}= 13.68 Hz[6,7].

$F_T \mapsto F'_T$	$F \mapsto F'$	$\nu_{\mathrm{(XA)B}}$	$\nu_{\mathrm{(NH)D}}$ (Hz)
$1 \mapsto 0$	$0 \mapsto 1$	$-J_{XB} + J_{XA}$	75.42
$1 \mapsto 1$	$0 \mapsto 1$	$-1/2J_{XB} + J_{XA}$	82.26
$1 \mapsto 2$	$0 \mapsto 1$	$1/2J_{XB} + J_{XA}$	95.94
$0 \mapsto 1$	$1 \mapsto 1$	$1/2J_{XB}$	6.84
$1 \mapsto 2$	$1 \mapsto 1$	J_{XB}	13.68

has a unique zero-field spectrum, which via averaging with an appropriate weight (see main text) is used to generate resultant spectra presented in the main text (Fig. 3).

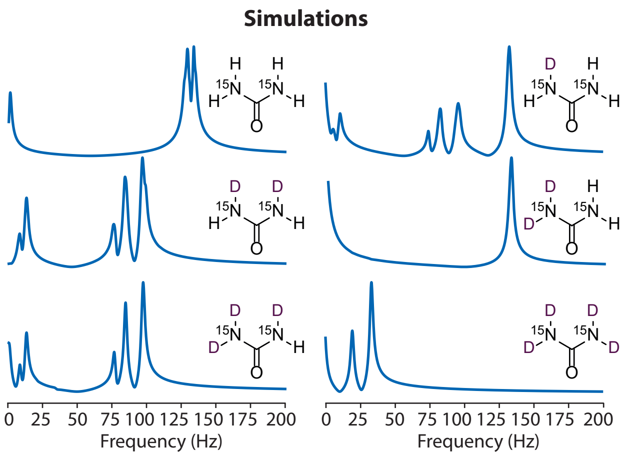

FIG. S2. Simulated zero-field J-spectra of deuterated [$^{15}N_2$]-urea isotopologues with corresponding structural formulas.

D. Details of chemical exchange simulations in zero-field

In urea, we distinguish two pH-dependent mechanisms for the proton exchange in the solution:

$$\begin{aligned} \mathrm{CO(NH_2)_2 + H^+} &\underset{k_d^C}{\overset{k_a^A}{\rightleftharpoons}} \mathrm{CO(NH_2)(NH_3^+)}, \\ \mathrm{CO(NH_2)_2 + OH^-} &\underset{k_a^D}{\overset{k_d^A}{\rightleftharpoons}} \mathrm{CO(NH_2)(NH^-) + H_2O}. \end{aligned} \tag{S5}$$

We label urea $CO(NH_2)_2$ as a subsystem A, $CO(NH_2)(NH_3^+)$ as a subsystem C, and $CO(NH_2)(NH^-)$ as a subsystem D and write kinetic equations for exchange reactions, assuming a first order kinetics in respect to acid, base, and urea:

$$\begin{aligned} \frac{d}{dt}[\mathrm{A}] &= -\,k_a^A[\mathrm{A}][\mathrm{H}^+] + k_d^C[\mathrm{C}], \\ \frac{d}{dt}[\mathrm{C}] &= k_a^A[\mathrm{A}][\mathrm{H}^+] - k_d^C[\mathrm{C}], \\ \frac{d}{dt}[\mathrm{A}] &= -\,k_d^A[\mathrm{A}][\mathrm{OH}^-] + k_a^D[\mathrm{D}], \\ \frac{d}{dt}[\mathrm{D}] &= k_d^A[\mathrm{A}][\mathrm{OH}^-] - k_a^D[\mathrm{D}], \end{aligned} \tag{S6}$$

where square brackets indicate concentration (or molar fraction). Above equations can be represented in a concise form:

$$\frac{d}{dt}\begin{pmatrix}[\mathrm{A}]\\ [\mathrm{C}]\\ [\mathrm{D}]\end{pmatrix} = \begin{pmatrix}\frac{1}{2}(-W_a^A - W_d^A) & \frac{1}{2}k_d^C & \frac{1}{2}k_a^D \\ W_a^A & -k_d^C & 0 \\ W_d^A & 0 & -k_a^D\end{pmatrix} \times \begin{pmatrix}[\mathrm{A}]\\ [\mathrm{C}]\\ [\mathrm{D}]\end{pmatrix}, \tag{S7}$$

where $W_a^A = k_a^A[\mathrm{H}^+]$ and $W_d^A = k_d^A[\mathrm{OH}^-]$.

To establish relation between the association and dissociation constants, we use the acid and base equilibrium constants:

$$\begin{aligned} K_a^{(1)} &= 10^{-pK_a^{(1)}} = \frac{k_a^A}{k_d^C}, \\ K_a^{(2)} &= \frac{10^{-14}}{K_b^{(2)}} = 10^{pK_b^{(2)}-14} = \frac{k_a^D}{k_d^A}, \end{aligned} \tag{S8}$$

where $pK_a^{(1)} = 0.1$, $pK_b^{(2)} = 13.83$[9,10]. For the dissociation constants of urea, we used $k_a^A = 9 \times 10^6\,\mathrm{s^{-1}mol^{-1}}$, $k_d^A = 2 \times 10^6\,\mathrm{s^{-1}mol^{-1}}$.[11]

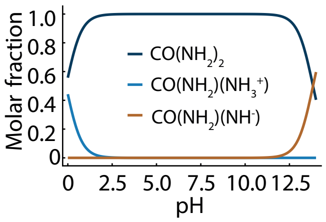

FIG. S3. Molar fraction of $CO(NH_2)_2$, $CO(NH_2)(NH_3^+)$, $CO(NH_2)(NH^-)$ in solution as a function of pH based on $pK_a^{(1)}$ and $pK_b^{(2)}$ values.

From now on, we follow the formalism established for calculating NMR spectra of molecules under a chemical exchange with solvents, presented in Ref. [12]. We construct a total-system state vector:

$$\rho(t) = \begin{pmatrix} \rho_A(t) \\ \rho_C(t) \\ \rho_D(t) \end{pmatrix},$$

where $\rho_A(t), \rho_C(t), \rho_D(t)$ are vectorized density matrices describing A, C and D subsystems (see, for example, Ref. [13]). From this point on, B indicates a spin-1/2 subsystem, describing an unpolarized state of the acidic proton H^+ or hydroxide OH^-. The evolution of the state-vector is governed by the spin superoperator $\mathbb{M}$:

$$\frac{d}{dt}\begin{pmatrix} \rho_A(t) \\ \rho_C(t) \\ \rho_D(t) \end{pmatrix} = \mathbb{M}\begin{pmatrix} \rho_A(t) \\ \rho_C(t) \\ \rho_D(t) \end{pmatrix} = \begin{pmatrix} \frac{1}{2}(-W_a^A - W_d^A)\mathbb{1}_A + \mathbb{L}_A & \frac{1}{2}k_d^C\mathbb{T}_B & \frac{1}{2}k_a^D\mathbb{D}_B \\ W_a^A\mathbb{D}_B & -k_d^C\mathbb{1}_C + \mathbb{L}_C & 0 \\ W_d^A\mathbb{T}_B & 0 & -k_a^D\mathbb{1}_D + \mathbb{L}_D \end{pmatrix}\begin{pmatrix} \rho_A(t) \\ \rho_C(t) \\ \rho_D(t) \end{pmatrix}, \tag{S9}$$

where $\mathbb{T}_B$ is a superoperator, corresponding to the average partial trace over the system B (average of partial traces removing each exchanging proton from the molecule) and $\mathbb{D}_B$ corresponds to a direct matrix product with the subsystem B. The $\mathbb{L}_i$ are spin superoperators, governing the evolution under the spin Hamiltonian $\mathcal{H}_i$, i.e., $\mathbb{L}_i = i(\mathbb{1} \otimes \mathcal{H}_i - \mathcal{H}_i \otimes \mathbb{1})$. The state-vector evolution is calculated by applying the propagation superoperator $\mathbb{M}$ to the state in a proceeding moment of time: $\rho(t + \Delta t) = e^{\mathbb{M}\Delta t}\rho(t)$, where the Δt is chosen to be smaller than the evolution timescale. At each time, the measurable magnetization M_z is obtained by evaluating $\langle M_z|\rho(t)\rangle$. The Fourier transform of the signal is then plotted and compared to the experimental results. The magnetization M_z is calculated taking into the account the pH-dependent molar fraction of each subsystem A, C, D, as shown in Fig. S3.

The simulations of the chemical exchange were performed using Matlab. The code for generating the partial trace and direct product matrices was adapted from the MOIN spin library[14,15]. To deal with memory restriction, while performing the calculation on large spin systems (up to 8 spin-1/2's), we use a Matlab function `expmv` that directly calculates action of the matrix exponential on the state vector without explicitly calculating the form of the matrix exponential[16,17].

The model presented here describes qualitatively the characteristics of zero-field urea spectra under the chemical exchange. Specifically, it predicts disappearance of high-frequency peaks, corresponding to ^{1}H-^{15}N coupling in acidic and basic conditions, as well as emergence of the narrow low-frequency structure related to coupling between ^{1}H-^{13}C under the same conditions. However, the simulations do not agree with experimental results in all details. For example, they predict much larger broadening of the signals at low and high pH than observed experimentally. This may be related to the limitations of our model, where the kinetics of exchange reactions are assumed to be first order with respect to urea concentration. This is true only for diluted urea samples, and at the concentration used in the study (8 M), the kinetics may deviate strongly from such a behavior. For example, the broadening of the resonances may be a result of clustering of urea in concentrated solutions. Furthermore, in the model, we assume complete depolarization of protons in the solutions, which holds true for moderate exchange rates and low-magnetic fields and may not be strictly correct in the case of fast exchange. This may be particularly pronounced in moderate fields, i.e., at the time right after the sample leaves prepolarization magnet, but prior to entering the zero-field region. Some shortcomings of the simulations (e.g., relying on the first-order kinetics) can be solved with the use of Monte Carlo simulations. At the same time, we believe that the simple exchange simulation approach presented here captures the essential features of the experimental results.

---

[1] Note that outside of the magnetic shield, the Earth's magnetic field coincides with solenoid guiding field effectively increasing the magnitude of transfer field. Inside the shield, the Earth's magnetic field is completely attenuated.

[2] M. C. Butler, M. P. Ledbetter, T. Theis, J. W. Blanchard, D. Budker, and A. Pines, The

Journal of Chemical Physics **138**, 184202 (2013).

[3] T. Theis, J. W. Blanchard, M. C. Butler, M. P. Ledbetter, D. Budker, and A. Pines, Chemical Physics Letters **580**, 160 (2013).

[4] A. Wilzewski, S. Afach, J. W. Blanchard, and D. Budker, Journal of Magnetic Resonance **284**, 66 (2017).

[5] S. Alcicek, P. Put, V. Kontul, and S. Pustelny, The Journal of Physical Chemistry Letters **12**, 787 (2021).

[6] P. W. Kuchel, C. Naumann, B. E. Chapman, D. Shishmarev, P. Håkansson, G. Bacskay, and N. S. Hush, Journal of Magnetic Resonance **247**, 72 (2014).

[7] O. Steinhof, E. J. Kibrik, G. Scherr, and H. Hasse, Magnetic Resonance in Chemistry **52**, 138 (2014).

[8] S. Afach, Spintrum (2018).

[9] I. M. Klotz and D. L. Hunston, The Journal of Physical Chemistry **75**, 2123 (1971).

[10] J. Bell, W. A. Gillespie, and D. B. Taylor, Transactions of the Faraday Society **39**, 137 (1943).

[11] R. L. Vold, E. Daniel, and S. Chan, Journal of the American Chemical Society **92**, 6771 (1970).

[12] D. Barskiy, M. Tayler, I. Marco-Rius, J. Kurhanewicz, D. Vigneron, S. Cikrikci, A. Aydogdu, M. Reh, A. Pravdivtsev, J.-B. Hövener, J. Blanchard, W. Teng, D. Budker, and A. Pines, Nature Communications **10**, 3002 (2019).

[13] T. Petrosky and I. Prigogine, Advances in Chemical Physics **99**, 1 (1997).

[14] A. N. Pravdivtsev and J.-B. Hövener, MOIN spin library (2021).

[15] A. N. Pravdivtsev and J.-B. Hövener, Chemistry–A European Journal **25**, 7659 (2019).

[16] N. J. Higham, Matrix exponential times a vector, MATLAB Central File Exchange (2021), https://github.com/higham/expmv.

[17] A. H. Al-Mohy and N. J. Higham, SIAM Journal on Scientific Computing **33**, 488 (2011).
